# Synthesis and Evaluation of In Vitro Antibacterial and Antitumor Activities of Novel N,N-Disubstituted Schiff Bases

**DOI:** 10.1155/2017/6257240

**Published:** 2017-06-21

**Authors:** Heng Luo, Yu-fen Xia, Bao-fei Sun, Li-rong Huang, Xing-hui Wang, Hua-yong Lou, Xu-hui Zhu, Wei-dong Pan, Xiao-dong Zhang

**Affiliations:** ^1^Beijing Chao-Yang Hospital, Capital Medical University, Beijing 100043, China; ^2^State Key Laboratory of Functions and Applications of Medicinal Plants, Guizhou Medical University, Guizhou 550014, China; ^3^Key Laboratory of Chemistry for Natural Products of Guizhou Province and Chinese Academy of Sciences, Guizhou 550014, China; ^4^Guizhou Medical University, Guizhou 550025, China

## Abstract

To get inside the properties of N,N-disubstituted Schiff bases, we synthesized three high-yielding benzaldehyde Schiff bases. We used the reaction between salicylaldehyde and different diamine compounds, including diamine, ethanediamine, and* o*-phenylenediamine, determining the structure of obtained molecules by nuclear magnetic resonance spectroscopy and electrospray ionization mass spectroscopy. We thus evaluated the microbicidal and antitumor activity of these compounds, showing that salicylaldehyde-hydrazine hydrate Schiff base (compound** 1a**) significantly inhibited the growth of* S. aureus*; salicylaldehyde-*o*-phenylenediamine Schiff base (compound** 1c**) displayed a strong capability to inhibit the proliferation of leukemia cell lines K562 and HEL. Moreover, we observed that the antibacterial action of** 1a** might be associated with the regulation of the expression of key virulence genes in* S. aureus*. Compound** 1c** resulted in a strong apoptotic activity against leukemia cells, also affecting the cell cycle distribution. Overall, our novel N,N-disubstituted Schiff bases possess unique antibacterial or antitumor activities that exhibit the potent application prospect in prophylactic or therapeutic interventions, providing new insights for developing new antibacterial and anticancer chemical agents.

## 1. Introduction

Recently, drug resistance of clinical bacteria and their pathogenicity as the major reasons caused the increasing rate of death in infectious diseases and tumor in humans because of the lack of effective drugs and methods for medical prevention and treatment [1–3]. Therefore, developing novel efficient antibacterial and antitumor agents is urgently needed based on new chemical compositions that have new structure with the natural products [4]. Schiff bases, discovered and named by the chemist Hugo Schiff, as a powerful candidate, exhibited various biological and physicochemical treated activities, antibacteria, anti-inflammation, and antitumor [5]. From chemical structure, we know that the carbonyl group (C=O) in some ketone or aldehyde-compounds is replaced by special functional groups, such as azomethine and/or imine group, to form series of special Schiff bases which are produced by the reaction of aldehydes or ketones with primary amines in the specific conditions [6, 7]. Previous studies have established the synthesis method and the biological activity assayed results showed that the presence of special functional groups (imine or azomethine subunits) is critical to their biological activities in various nonnatural, natural, and natural-derived compounds [8].

There are evidences confirming that salicylaldehyde Schiff bases, obtained from the condensation of the salicylaldehyde and its derivatives in alkaline grind solution [8, 9], showed a better carrying oxygen ability and catalysis of mimic enzymes due to their structure similar to the porphyrin and phthalocyanine rings, which displayed a great anticancer, anti-inflammatory, antibacterial, and antiviral activity. N,N-Disubstituted Schiff bases are a series of easy flowed electronic bridge structures, which can chelate with metal ions to form a flat rigid *π* conjugate structure with fluorescence characteristics when aromatic rings were introduced to the salicylaldehyde moiety [9–11]. However, a diamine can react with salicylaldehyde by condensation reaction in alkaline grind solution to produce N,N-disubstituted Schiff bases [8]; their biological activity has been attributed to the presence of the (–CONHN=CH–) moiety. Some hydrazide-hydrazone derivatives possessed a broad range of biological activities in vivo and in vitro including analgesic, anticonvulsant [12], antidepressant [13], antimicrobial [14, 15], antitumor [16, 17], and anti-inflammatory [18] activities, while little available research reported the biological activity and mechanism of N,N-disubstituted Schiff bases. In this paper, we synthesized and characterized three N,N-disubstituted hydrazone Schiff bases, thus assessing their antibacterial and antitumor activities in vitro. Results will validate the antibacterial and antitumor efficacy and large differences of structure-activity relationships of N,N-disubstituted hydrazone Schiff bases in vitro and also provided the chance for developing the potential antibacterial agents from the Schiff bases.

## 2. Materials and Methods

### 2.1. General

All equipment, spectrometer and column chromatography, and chemical or biological agents used in this study were the same as previously published paper [19]. Cell lines PC3, MDA, WM9, BPH1, K562, and HEL were a gift obtained from the Sunnybrook Research Center in Canada.

### 2.2. Synthesis Procedure for N,N-Disubstituted Schiff Bases

Compounds** 1a**,** 1b,** and** 1c** were synthesized according to the report of Przybylski et al. [11]. In brief, salicylaldehyde (0.01 mol, 2 eq) was injected in 50 mL anhydrous ethanol in a round bottom flask and then added to 85% hydrazine hydrate (0.005 mol, 1 eq). The reaction mixture was then refluxed for 7 h at 80°C under Ar_2_ protection and detected by thin layer chromatography (TLC) assay. After cooling, the obtained product was filtered and then washed with cold ethanol and dried. Recrystallization was done using ethanol. Compounds** 1b** and** 1c** were prepared by adding ethanediamine and* o-*phenylenediamine.

#### 2.2.1. N,N′-Di-(2-hydroxy)-benzyl-hydrazine (**1a**)

Green needle crystal, yield 32.7%; ESI-MS:* m/z* 264.1 [M+Na]^+^; ^1^H-NMR (CD_3_OD, 400 MHz) *δ* (ppm): 6.95 (m, 1H, 5-H), 6.96 (m, 1H, 5′-H), 7.02 (d, 1H, 3-H,* J* = 5.6 Hz), 7.04 (d, 1H, 3′-H,* J* = 5.6 Hz), 7.34 (m, 1H, 4′-H), 7.37 (m, 1H, 4-H), 7.39 (m, 1H, 6′-H), 7.40 (m, 1H, 6-H); ^13^C-NMR (CDCl_3_, 100 MHz) *δ* (ppm): 164.7, 159.7, 133.4, 132.5, 119.7, 117.2, 117.1.

#### 2.2.2. N,N′-Di-(2-hydroxy)-benzyl-ethylenediamine (**1b**)

Bright yellow crystal plate, yield = 42.3%; ESI-MS:* m/z* 291.0 [M+Na]^+^; ^1^H-NMR (CDCl_3_, 400 MHz) *δ* (ppm): 3.93 (s, 4H, 1′′, 2′′-CH_2_), 6.83 (m, 1H, 3′-H), 6.85 (m, 1H, 3-H), 6.92 (m, 1H, 5-H), 6.94 (m, 1H, 5′-H), 7.21 (m, 1H, 4-H), 7.23 (m, 1H, 4′-H), 7.29 (m, 1H, 6′-H), 7.30 (m, 1H, 6-H), 8.35 (s, 2H, N=CH); ^13^C-NMR (CDCl_3_, 100 MHz) *δ* (ppm): 166.4, 160.9, 132.4, 131.4, 118.6, 118.5, 116.9, 59.7.

#### 2.2.3. N,N′-Salicylaldehyde-*o*-phenylenediamine (**1c**)

Orange needle crystal, yield = 21.8%; ESI-MS:* m/z* 339.0 [M+Na]^+^; ^1^H-NMR (CDCl_3_, 400 MHz) *δ* (ppm): 6.91 (m, 1H, 5-H), 6.93 (m, 1H, 5′-H), 7.04 (m, 1H, 3-H), 7.06 (m, 1H, 3′-H), 7.23 (m, 1H, 3′′-H), 7.25 (m, 1H, 6′′-H), 7.33–7.39 (m, 6H, 4, 4′, 6, 6′, 4′′, 5′′-H), 8.63 (s, 2H, N=CH); ^13^C-NMR (CDCl_3_, 100 MHz) *δ* (ppm): 163.7, 161.3, 142.5, 133.4, 132.3, 127.7, 119.7, 119.2, 118.9, 117.5.

### 2.3. Antibacterial Activity Assay

The antibacterial activity in vitro of the compounds was assessed in vitro by turbidimetric assays [19, 20]. The minimum inhibitory concentration (MIC) value was determined with broth microdilution method [21].

### 2.4. *In Vitro* Gene Expression

The methods were the same as described previously [19, 21].

### 2.5. Antitumor Activity Assay

#### 2.5.1. Cell Cultures

Cell cultures (i.e., PC3, MDA, WM9, BPH1, K562, and HEL) were incubated at 37°C and 5% CO_2_ as monolayer in RPMI 1640 medium (Hyclone, Germany) containing 10% heat inactivated fetal bovine serum (Hyclone).

#### 2.5.2. Antitumor Activity Assay

Antitumor activity was evaluated by performing the MTT assay [17]. Briefly, PC3, MDA, WM9, BPH1, K562, and HEL cells were seeded in 96-well microculture plates at the density of 5 × 10^3^ cells/well and incubated for 24 h to allow cell adhesion. Cells were then treated with various concentrations of assayed compounds for 48 h and then observed with an inverted fluorescence microscope (Nikon, Japan). MTT (20 *μ*L of 5 mg/mL solution) was added to each well and incubated at 37°C for additional 4 h. All medium was then removed and added 200 *μ*L Tris-DMSO solution. Plates were lightly shaken up for dissolving the mixture to measure the absorbance at 570 nm using an ELISA plate reader.

#### 2.5.3. Cell Apoptosis Assay

Cells apoptosis was also evaluated using flow cytometer based on the reported methods [16, 17]. Apoptotic cells were defined as annexin V positive control. The treated cell was trypsinized, washed using PBS solution, transferred to microcentrifuge tubes for centrifugation at 1000 rpm for 5 minutes, and resuspended in binding buffer. Propidium iodide (Becton Dickinson Pharmingen, Franklin Lakes, NJ, USA) was added to the cells to a 20 *μ*g/mL of final concentration. The mixture was transferred to a 96-well plate to analyze induced apoptosis by flow cytometer (Becton Dickinson Biosciences, Franklin Lakes, NJ, USA).

#### 2.5.4. Cell Cycle Assay

Treated cells were washed in PBS solution, trypsinized, and transferred onto microtubes for centrifugation at 2000 rpm for 3 min. The cell pellet was fixed by adding ice-cold ethanol to avoid cell clumping. After one hour at 4°C, ethanol solution in the cells was removed by centrifuging at 1500 rpm for 5 min. The cell pellet was then resuspended using PBS solution containing 1 *μ*g/mL RNase and incubated at 37°C for 30 minutes and added to a final concentration of 20 *μ*g/mL propidium iodide. The mixture was transferred into a 96-well plate to analyze the propidium iodide signal intensity using flow cytometer with FACSArray (BD Biosciences, Franklin Lakes, NJ, USA). The signal intensity was determined by the percentage of cells at G_0_, G_1_, and S phases.

#### 2.5.5. Statistical Analysis

SPSS 18.0 software was used for analyzing the data and reported results indicated the mean ± SD of three experiments. For all the experiments, the statistical significance of difference between each group was determined by one-way ANOVA followed by Student's* t*-test. The statistical significance of difference between every two groups was investigated with LSD method. *P* < 0.05 was defined as significant and *P* < 0.01 was considered extremely significant. Dates were presented as the mean ± SEM of three assays.

## 3. Results

### 3.1. Chemistry

**Scheme 1:**
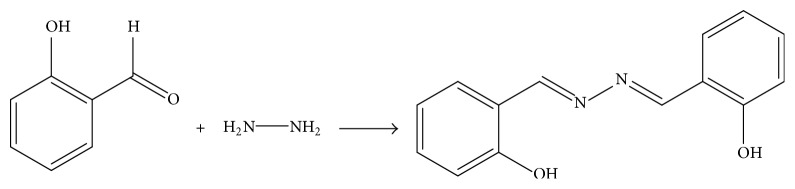
The synthesis and chemical structure of salicylaldehyde-hydrazine hydrate Schiff base (**1a**).

**Scheme 2:**
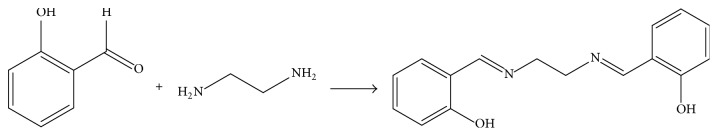
The synthesis and chemical structure of salicylaldehyde-ethylenediamine Schiff base (**1b**).

**Scheme 3:**
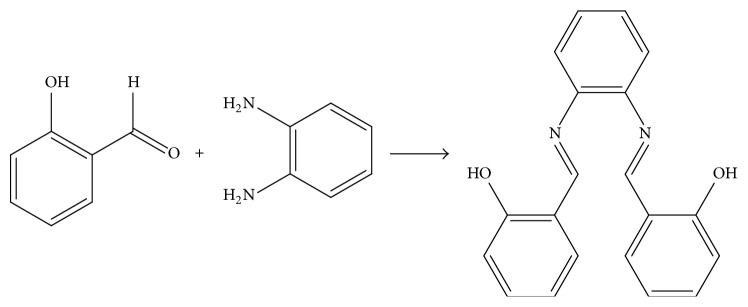
The synthesis and chemical structure of salicylaldehyde-*o-*phenylenediamine Schiff base (**1c**).

Three N,N-disubstituted Schiff bases (**1a**,** 1b,** and** 1c**) were produced according to the condensation reaction between salicylaldehyde and different diamine compounds, including diamine (Scheme 1), ethanediamine (Scheme 2), and* o*-phenylenediamine (Scheme 3), in the presence of an alkali [11]. Three diamine compounds (1 eq) and salicylaldehyde (1 eq) were added to anhydrous ethanol with stirring and refluxing to produce the three target compounds. All reactions were determined by TLC assay. The structures of compounds** 1a–1c** were determined with ESI-MS data and NMR.

### 3.2. Antibacterial Activity of N,N-Disubstituted Schiff Bases

A preliminary evaluation of 100 *μ*mol/L purified N,N-disubstituted Schiff bases was assayed to determine the antibacterial activity by assessing the antigrowth capability to several clinical pathogenic bacteria. Results obtained are summarized in Table 1. We observed that the growth of* S. aureus* was inhibited significantly by compound** 1a**, with an extent similar to the positive control. Moreover, compounds** 1a** and** 1b** both exhibited slight inhibitory activity against* E. coli* (inhibition < 50% bacterial cell growth). We also observed that the three analyzed compounds could selectively suppress the growth of* A. baumannii*,* K. pneumonia*, and* P. aeruginosa* in a very slight extent. However, we decided to further investigate the antibacterial properties of compound** 1a** that exhibited more than 50% bacterial cell growth inhibition at the concentration of 100 *μ*mol/L. We thus confirmed the antibacterial activity by determining the minimum inhibitory concentration (MIC) values.

**Figure 1:**
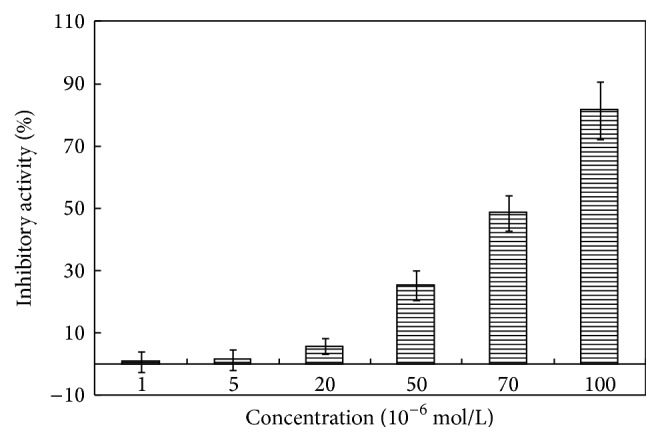
Evaluation of the inhibitory activity of salicylaldehyde-hydrazine hydrate Schiff base (**1a**) against* S. aureus* in vitro. Various concentrations of compound** 1a** were added to 96-well microculture plates containing the* S. aureus* strain ATCC 25923 at concentration of 10^5^ CFU/mL in Luria Broth. The absorbance of every well at 450 nm was assayed in an ELISA plate reader after shaking on a vibrating platform at 37°C for 8 h. The inhibition ratio (%) was determined as reported in Materials and Methods. Values are mean ± standard deviation of three independent experiments.

To this aim, the bacteria were cultured at 37°C for 8 hours in LB medium containing different concentrations of compound** 1a** in order to investigate whether the synthesis compounds demonstrated antibacterial activity at a concentration < 100 *μ*mol/L. The MIC value of each obtained compound for the bacterial growth was defined as the lowest concentration of the compound that reduced the growth by 1% compared to the control [19]. Obtained results indicated that salicylaldehyde-hydrazine hydrate Schiff base (compound** 1a**) displayed an activity for inhibiting the growth of* S. aureus* in a concentration-dependent manner (Figure 1), with a MIC value of 9.75 ± 1.02 *μ*mol/L, which was similar to the positive control Streptomycin.

### 3.3. Effect of the Compound** 1a** on Expression of Virulence Genes in* S. aureus*

**Figure 2:**
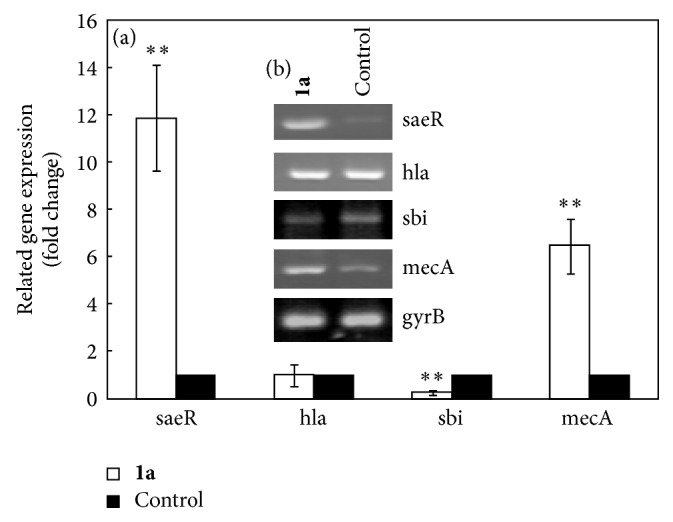
The compound** 1a** affects* S. aureus* virulence genes expression in vitro. (a) Fold changes of the expression of the related virulence genes were determined by real-time RT-PCR. (b) The transcript expression level of the genes was investigated using semiquantitative RT-PCR. Data are normalized to the transcript abundance of* gyrB *gene. Values are mean ± standard deviation of three independent experiments. ^*∗∗*^*P* < 0.01.

Salicylaldehyde-hydrazine hydrate Schiff base (**1a**) showed the better inhibition to the growth of* S. aureus*. To further investigate the action of this compound and its effect on the expression of associated virulence factors, we cultured* S. aureus* ATCC 25923 with the treatment of a sublethal dose of the compound (50 *μ*mol/L) for 8 h; the expressional level in mRNA (transcript abundance) of the key virulence factors* hla*,* sbi*,* saeR*, and* mecA* was determined (*gyrB* housekeeping gene was used as control) by RT-PCR (Figure 2(a)) and semiquantitative RT-PCR methods (Figure 2(b)). Compound** 1a** resulted in a significant (*P* < 0.01) induction of the expression of* saeR* and* mecA* genes, with 12-fold and 6-fold higher expression than the negative control, respectively. Besides, compound** 1a** downregulated the expression of* sbi* gene (*P* < 0.01), about 5-fold lower than the negative control. Finally, no changes were observed in the expression of* hla* genes.

### 3.4. Antitumor Activity of N,N-Disubstituted Schiff Bases

**Figure 3:**
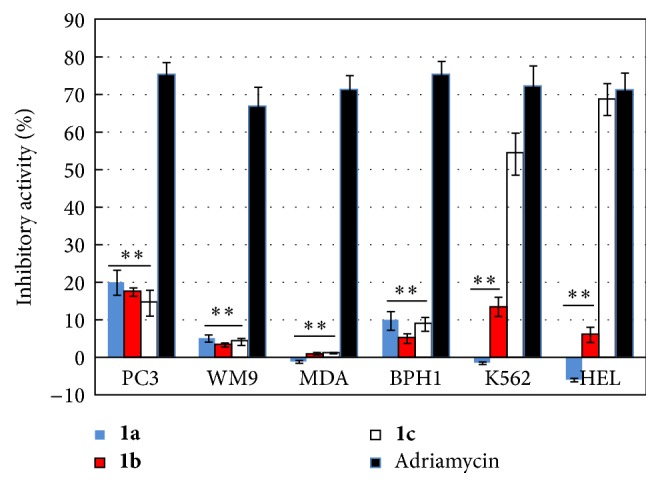
Antitumor activity in vitro of compounds** 1a**,** 1b,** and** 1c **assayed at the concentration of 5 *μ*mol/L. Cancer cell survival was assayed by MTT method. The results represent the mean ± standard deviation of three independent experiments. ^*∗∗*^*P* < 0.01.

**Figure 4:**
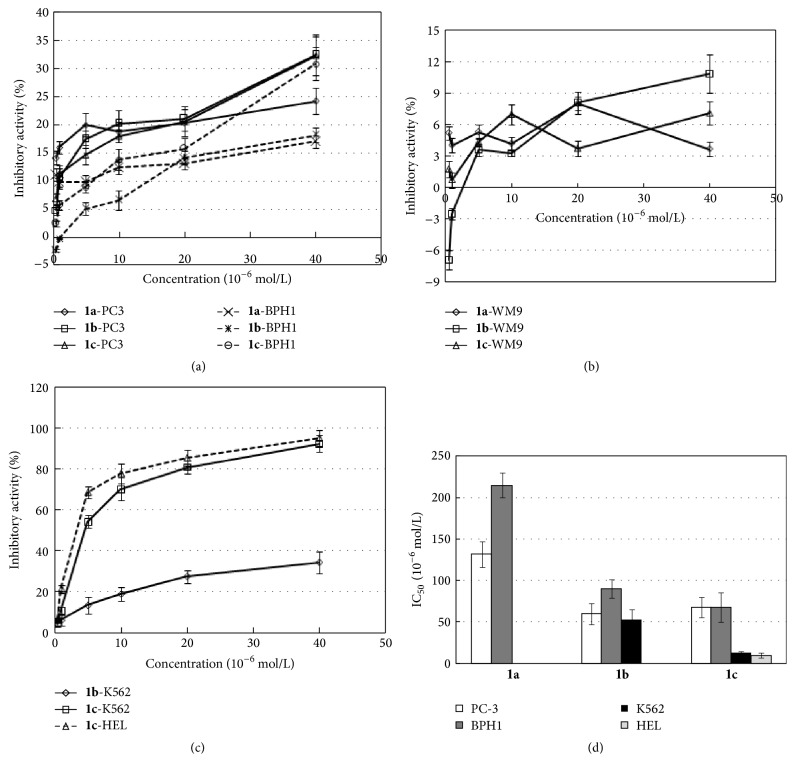
Concentration-inhibition curves of the active compounds against the proliferation of prostate ((a) PC3 and BPH1 cells), melanoma ((b) WM9 cells), and leukemia ((c) K562 and HEL cells) cell lines in vitro. Different concentrations of tested compounds were added to 96-well microculture plates and cells were incubated for 48 h at 37°C. The inhibition ratio (%) was calculated as described in Materials and Methods.

The cytotoxic activity of the three N,N-disubstituted Schiff bases was evaluated on several tumor cell lines by assaying various concentrations of compounds. Using MTT assay determined the cell viability (Figure 3), and we further analyzed the concentration-inhibition curves, reported in Figure 4, in order to calculate the IC_50_ values (Table 2). Our results showed that the three investigated Schiff bases exhibited different inhibitory ability on the growth of several human cell lines. Compounds** 1a**,** 1b,** and** 1c** could moderately inhibit the proliferation of human prostate cancer cell line (PC3) and prostate mesenchymal cell line (BPH1) but failed to detect significant effects in the cytotoxic activity on melanoma cell line (WM9) and breast cancer cell line (MDA) at concentration of 5 *μ*mol/L (*P* < 0.01, compared with control). Interesting, only salicylaldehyde-*o*-phenylenediamine Schiff base (compound** 1c**) displayed a higher inhibitory activity on the growth of the two leukemia cell lines K562 and HEL, at same concentration, with IC_50_ values of 11.95 ± 2.36 *μ*mol/L and 9.72 ± 2.56 *μ*mol/L, respectively. The IC_50_ value was determined with the semilogarithmic dose-response curves. The cytotoxic activity of compounds** 1a**,** 1b,** and** 1c** on PC3 (Figure 4(a)), BPH1 (Figure 4(a)), K562 (Figure 4(c)), and HEL (Figure 4(c)) cell lines was increased with the increase of assayed concentrations of the compounds, indicating the dose-dependent trend of the inhibitory response. However, the same trend was not observed in the inhibitory activity of the compounds on the WM9 cell (Figure 4(b)).

**Figure 5:**
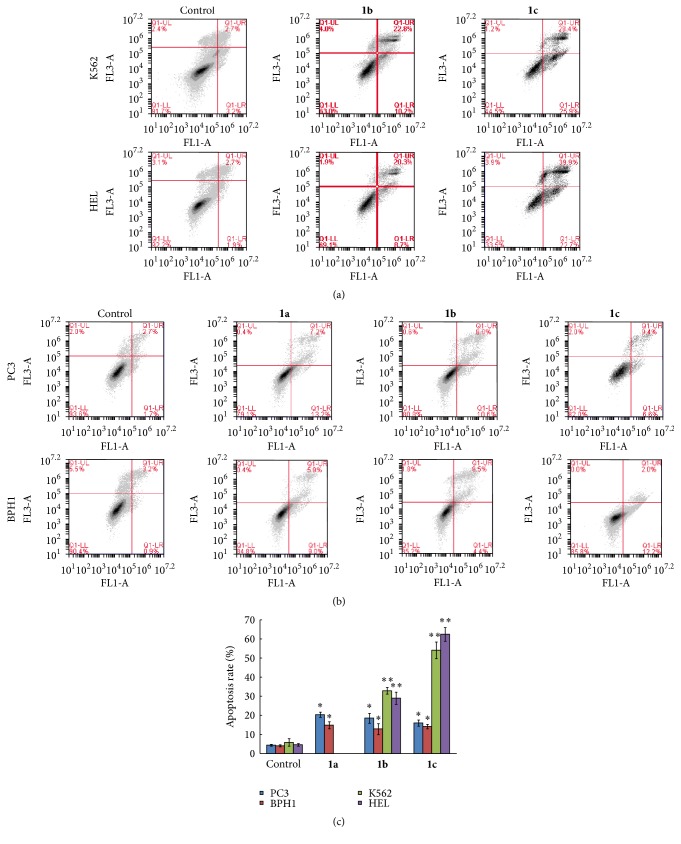
Evaluation of the apoptosis induced by the three N,N-disubstituted Schiff bases on two leukemia cell lines (a) and two prostate cell lines (b) using the annexin V-FITC/IP staining, followed by flow cytometer analysis. (c) Comparison of the apoptosis induced by compounds** 1a**,** 1b,** and** 1c**. Histograms represent annexin V-FITC/IP stained cells cultured in the presence of 5 *μ*mol/L of tested compounds. Data showed the percentage of late induced apoptotic cells (upper right quadrant) and represent the mean ± standard deviation of three independent experiments, each performed in duplicate. ^*∗*^*P* < 0.05; ^*∗∗*^*P* < 0.01.

All cells treated with 5 *μ*mol/L of each compound for 48 h were also analyzed using a flow cytometer, in order to observe apoptosis (Figure 5). Compared to untreated cells (Figure 5(c)), K562 and HEL cells treated with compounds** 1b** and** 1c** showed apoptosis rates significantly increased (Figure 5(a)). Furthermore, compound** 1c** was showed to be able to induce high levels of apoptosis in two leukemia cell lines. However, very low apoptosis rates were observed in PC3 and BPH1 cells (Figure 5(b)).

### 3.5. Effects of N,N-Disubstituted Schiff Bases on Cell Cycle

**Figure 6:**
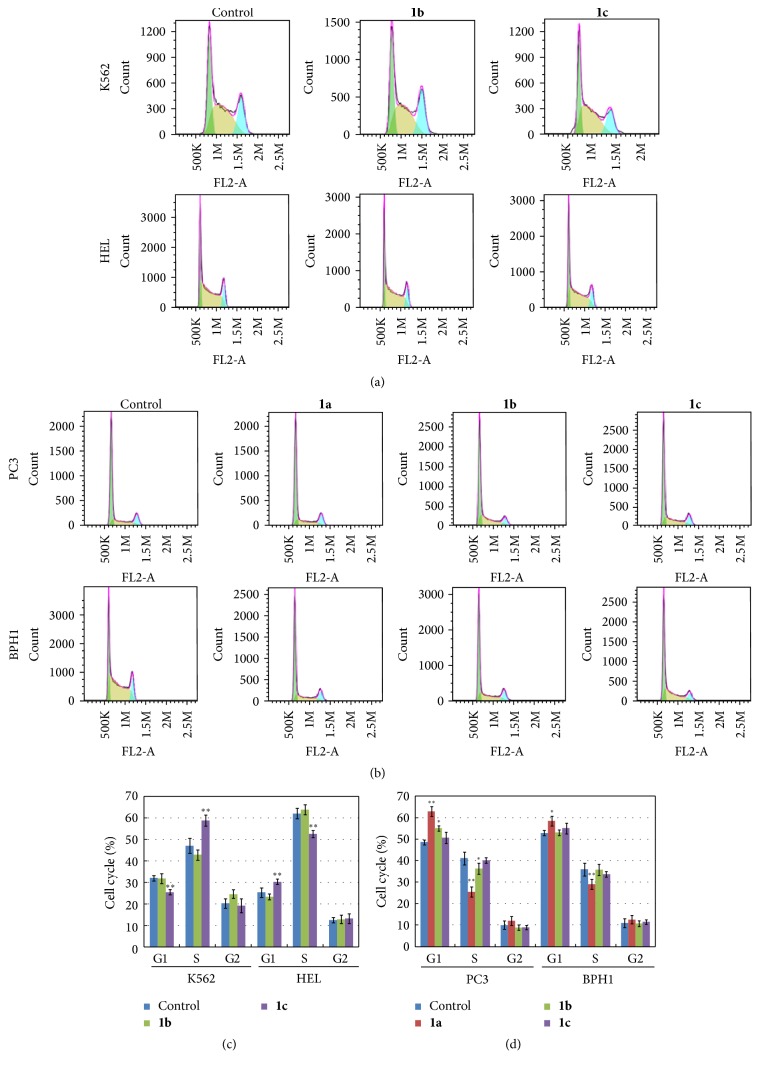
Effects of the three N,N-disubstituted Schiff bases on the cell cycle of leukemia and prostate cells. K562 and HEL leukemia cell lines (a) and one prostate cancer cell line PC3 and prostate mesenchymal cell line BPH1 (b) were used for assaying the cell cycle change by the treating with the active compounds, and then, the date were analyzed to obtain the more intuitive results (c and d). Compounds** 1a**,** 1b,** and** 1c** were assayed at the concentration of 20 *μ*mol/L. Data represent the mean ± standard deviation of three independent experiments. ^*∗*^*P* < 0.05; ^*∗∗*^*P* < 0.01.

Effects of active compounds on cell cycle distribution in leukemia (Figure 6(a)) and prostate (Figure 6(b)) cells were evaluated using flow cytometer. After the incubation with 20 *μ*mol/L of the compounds for 48 hours, cells were harvested and analyzed. Results showed that cells distribution in G1 and S phases was affected in the two K562 and HEL leukemia cells treated with compound** 1c** (*P* < 0.01, Figure 6(c)). However, compound** 1b** did not induce changes in the two cell lines. Compound** 1a** was found to significantly increase the G1 phase of prostate PC3 (*P* < 0.01) and BPH1 cells (*P* < 0.05) along with a reduction of the number of cells in S phase (Figure 6(d)). Compound** 1b** could only slightly change the G1 and S phases of PC3 cell (*P* < 0.05) but has no significant effect on BPH1 cells. Finally, the compound** 1c** induced no significant changes in cell cycle profile of the PC3 and BPH1 cells.

## 4. Discussion

In the present study, we synthesized three novel N,N-disubstituted Schiff bases and evaluated their properties as antibacterial and antitumor agents. Overall, we found that the salicylaldehyde-hydrazine hydrate Schiff base (compound** 1a**) exhibited the best antibacterial feature, with an inhibitory activity against* S. aureus* proliferation similar to the positive control. On the other hand, the salicylaldehyde-*o-*phenylenediamine Schiff base (compound** 1c**) showed the higher inhibitory activity on the proliferation of leukemia cell lines (K562 and HEL). These results support the idea of N,N-di-substitution Schiff bases as a promising drug candidate for treating infections caused by* S. aureus* or leukemia in human. More in detail, the gene expression assay indicated that compound** 1a** could regulate the expression of some genes involved in virulence factor, especially for* saeR* gene. It is clear that further studies will be necessary to elucidate the mechanism of action of N,N-disubstituted Schiff bases as antibacterial agents.

The strong inhibitory effect of compound** 1a** on the growth of* S. aureus* suggests that the linked hydrazine might significantly improve the antibacterial activity of N,N-disubstituted Schiff bases. Similarly, the phenyl group was inserted to form the salicylaldehyde-*o*-phenylenediamine Schiff base (compound** 1c**) that exhibited a potent inhibition on the growth of leukemia cells, which may play an essential role in this selective anticancer activity. However, further studies are required. Some previous studies have elucidated the mechanism of action related to the antimicrobial activity of N,N-disubstituted Schiff bases, showing the involvement of the regulation of genes associated with virulence factors [19]. Transcript of* saeR* gene, a key member of the virulence regulatory system saeR/S that plays an important role in the development of staphylococcal skin lesions in mice [22], was upregulated about 12-fold in* S. aureus* following the incubation with** 1a** with respect to the control. Besides, we have also analyzed the expression of other virulence genes* sbi*,* hla,* and* mecA* at the transcriptional level. Gene* hla* encodes the *α*-hemolysin, which is essential for* S. aureus* and causes skin infections diseases in both animal and human [23]; gene* sbi* encodes for a crucial immunomodulatory protein in the complement evasion [24]; gene* mecA* encodes for the altered penicillin-binding protein 2a conferring resistance to *β*-lactam antibiotic [25].

Our results demonstrate that the transcriptional expression of several virulence genes was upregulated by compound** 1a**. The reduction of transcript levels of other virulence genes of* S. aureus* involved in the saeR/S virulence regulatory system indicates that compound** 1a** may regulate in an intricate manner a grown number of* S. aureus* virulence genes, supporting the hypothesis that the antigrowth activity of Schiff bases against* S. aureus* may associate with the up- or downregulation on the expression of related virulence gene. Our results also indicate that only the compound** 1c** possesses a slight inhibitory activity against prostate cells along with a strong capability to inhibit leukemia cell proliferation, thus representing a novel powerful candidate as antitumor agent.

## Figures and Tables

**Table 1:** Antibacterial activity of the three compounds (100 *μ*mol/L) against six strains in vitro.

Compounds	Inhibition rate (%)					
	*E. coli * ATCC 25922	*B. subtilis * ATCC 6051	*S. aureus * ATCC 25923	*A. baumannii * ATCC BAA-1710D	*K. pneumonia * ATCC BAA-1705	*P. aeruginosa * ATCC 39324
**1a**	11.33 ± 7.24	Inactive	91.51 ± 7.98^*∗∗*^	8.51 ± 1.35	5.03 ± 2.36	Inactive
**1b**	16.55 ± 4.22	Inactive	Inactive	10.05 ± 2.46	8.07 ± 1.23	1.38 ± 1.26
**1c**	0.55 ± 2.19	Inactive	Inactive	14.40 ± 3.59	13.54 ± 4.58	8.65 ± 3.58
Ampicillin	99.34 ± 3.26	—	—	—	—	—
Streptomycin	—	94.12 ± 6.56	97.05 ± 4.24	—	—	—
Kanamycin	—	—			87.34 ± 3.19	—
Chloramphenicol	—	—	—	82.43 ± 9.29	—	86.65 ± 7.37

Values are mean ± standard deviation of three independent experiments. The bacteria were seeded in 96-well microplates at concentration of 1 × 10^5^ CFU/mL in Luria Broth medium. Tested compounds and positive controls were added to a final concentration of 100 *μ*mol/L. Inhibiting growth of the bacteria was determined at 450 nm using an ELISA plate reader after shaking on a vibrating platform at 37°C for 8 h. ^*∗∗*^*P* < 0.01 compared with the control.

**Table 2:** IC_50_ values of compounds **1a**, **1b,** and **1c** on cancer cell lines.

Cancer cell lines	IC_50_ (*μ*mol/L)			
	**1a**	**1b**	**1c**	Adriamycin
PC3	131.26 ± 15.36^*∗∗*^	59.78 ± 12.13^*∗∗*^	67.24 ± 13.14^*∗∗*^	8.06 ± 1.42
BPH1	214.61 ± 14.25^*∗∗*^	90.03 ± 11.36^*∗∗*^	67.39 ± 15.89^*∗∗*^	11.36 ± 1.14
K562	—	52.22 ± 10.39^*∗∗*^	11.95 ± 2.36	4.56 ± 0.88
HEL	—	—	9.72 ± 2.56	3.12 ± 0.32

Cell lines include the following: human prostate cancer cell line (PC3), human prostate mesenchymal cell line (BPH1), and human leukemia cell lines (K562 and HEL). Cell viability (%) was determined by MTT assay to calculate the IC_50_. All the assayed compounds were dissolved in DMSO, with a final concentration of DMSO was less than 0.1%. Control cells were treated only with the medium containing 0.1% DMSO. Values were mean ± standard deviation of three independent experiments.^*∗∗*^*P* < 0.01.

## References

[1] Da Silva C. M., Da Silva D. L., Modolo L. V. (2011). Schiff bases: a short review of their antimicrobial activities. *Journal of Advanced Research*.

[2] Baquero F. (1997). Gram-positive resistance: challenge for the development of new antibiotics. *Journal of Antimicrobial Chemotherapy*.

[3] Alekshun M. N., Levy S. B. (2007). Molecular mechanisms of antibacterial multidrug resistance. *Cell*.

[4] Rice L. B. (2006). Unmet medical needs in antibacterial therapy. *Biochemical Pharmacology*.

[5] Schiff H. (1864). Mittheilungen aus dem Universitätslaboratorium in Pisa: Eine neue Reihe organischer Basen. *Justus Liebigs Annalen der Chemie*.

[6] Bringmann G., Dreyer M., Faber J. H. (2004). Ancistrotanzanine C and related 5,1′- and 7,3′-coupled naphthylisoquinoline alkaloids from Ancistrocladus tanzaniensis. *Journal of Natural Products*.

[7] De Souza A. O., Galetti F. C. S., Silva C. L. (2007). Antimycobacterial and cytotoxicity activity of synthetic and natural compounds. *Quimica Nova*.

[8] Sharma S., Jain A., Aggarwal A., Gill N. (2012). Synthesis, Characterization and pharmacological evaluation of novel schiff bases of imide moiety. *Journal of Medical Sciences*.

[9] Guo Z., Xing R., Liu S. (2007). Antifungal properties of Schiff bases of chitosan, N-substituted chitosan and quaternized chitosan. *Carbohydrate Research*.

[10] Dhar DNTaploo CL. (1982). *Schiff bases and their applications*.

[11] Przybylski P., Huczynski A., Pyta K., Brzezinski B., Bartl F. (2009). Biological properties of schiff bases and azo derivatives of phenols. *Current Organic Chemistry*.

[12] Ragavendran J. V., Sriram D., Patel S. K. (2007). Design and synthesis of anticonvulsants from a combined phthalimide-GABA-anilide and hydrazone pharmacophore. *European Journal of Medicinal Chemistry*.

[13] Ergenc N., Gunay N. S. (1998). Synthesis and antidepressant evaluation of new 3-phenyl-5-sulfonamidoindole derivatives. *European Journal of Medicinal Chemistry*.

[14] Vicini P., Zani F., Cozzini P., Doytchinova I. (2002). Hydrazones of 1,2-benzisothiazole hydrazides: synthesis, antimicrobial activity and QSAR investigations. *European Journal of Medicinal Chemistry*.

[15] Jayabharathi J., Thangamani A., Padmavathy M., Krishnakumar B. (2007). Synthesis and microbial evaluation of novel N(1)-Arilidene-N(2)-t(3)- methyl-r(2), c(6)-diaryl-piperidin-4-one azine derivatives. *Medicinal Chemistry Research*.

[16] Zhang H.-Z., Drewe J., Tseng B., Kasibhatla S., Cai S. X. (2004). Discovery and SAR of indole-2-carboxylic acid benzylidene-hydrazides as a new series of potent apoptosis inducers using a cell-based HTS assay. *Bioorganic and Medicinal Chemistry*.

[17] El-Hawash S. A. M., Abdel Wahab A. E., El-Demellawy M. A. (2006). Cyanoacetic acid hydrazones of 3-(and 4-)acetylpyridine and some derived ring systems as potential antitumor and anti-HCV agents. *Archiv der Pharmazie*.

[18] Todeschini A. R., de Miranda A. L. P., da Silva K. C. M., Parrini S. C., Barreiro E. J. (1998). Synthesis and evaluation of analgesic, antiinflammatory and antiplatelet properties of new 2-pyridylarylhydrazone derivatives. *European Journal of Medicinal Chemistry*.

[19] Xia L., Xia Y.-F., Huang L.-R. (2015). Benzaldehyde Schiff bases regulation to the metabolism, hemolysis, and virulence genes expression in vitro and their structure-microbicidal activity relationship. *European Journal of Medicinal Chemistry*.

[20] Huang L., Luo H., Li Q. (2015). Pentacyclic triterpene derivatives possessing polyhydroxyl ring A inhibit Gram-positive bacteria growth by regulating metabolism and virulence genes expression. *European Journal of Medicinal Chemistry*.

[21] Huang L.-R., Hao X.-J., Li Q.-J. (2016). 18*β*-Glycyrrhetinic Acid Derivatives Possessing a Trihydroxylated A Ring Are Potent Gram-Positive Antibacterial Agents. *Journal of Natural Products*.

[22] Nygaard T. K., Pallister K. B., Ruzevich P., Griffith S., Vuong C., Voyich J. M. (2010). Saer binds a consensus sequence within virulence Gene promoters to advance USA300 pathogenesis. *Journal of Infectious Diseases*.

[23] Kennedy A. D., Wardenburg J. B., Gardner D. J. (2010). Targeting of alpha-hemolysin by active or passive immunization decreases severity of USA300 skin infection in a mouse model. *Journal of Infectious Diseases*.

[24] Smith E. J., Visai L., Kerrigan S. W., Speziale P., Foster T. J. (2011). The Sbi protein is a multifunctional immune evasion factor of *Staphylococcus aureus*. *Infection and Immunity*.

[25] Long D. R., Mead J., Hendricks J. M., Hardy M. E., Voyich J. M. (2013). 18*β*-glycyrrhetinic acid inhibits methicillin-resistant Staphylococcus aureus survival and attenuates virulence gene expression. *Antimicrobial Agents and Chemotherapy*.

